# The mother of all endocytosis

**DOI:** 10.7554/eLife.01738

**Published:** 2013-11-26

**Authors:** David E Clapham

**Affiliations:** 1 **David E Clapham** is in the Howard Hughes Medical Institute, Department of Cardiology, Boston Children’s Hospital, Boston, United States and the Department of Neurobiology, Harvard Medical School, Boston, United Statesdclapham@enders.tch.harvard.edu

**Keywords:** endocytosis, palmitoylation, patch clamp, permeability transition pore, None

## Abstract

Massive endocytosis is initiated by a series of steps that involve a sudden influx of calcium ions, changes in mitochondria, and modification of surface proteins by lipids. A better understanding of this process could lead to new approaches to reducing the tissue damage that is caused by heart attacks.

**Related research articles** Hilgemann DW, Fine M, Linder ME, Jennings BC, Lin M-J. 2013. Massive endocytosis triggered by surface membrane palmitoylation under mitochondrial control in BHK fibroblasts. *eLife* **2**:e01293. doi: 10.7554/eLife.01293

Lin M-J, Fine M, Lu J-Y, Hofmann SL, Frazier G, Hilgemann DW. 2013. Massive palmitoylation-dependent endocytosis during reoxygenation of anoxic cardiac muscle. *eLife* **2**:e01295. doi: 10.7554/eLife.01295

**Image** Micrograph of a patch clamp experiment with a BHK cell (the circular structure at the bottom of the image)

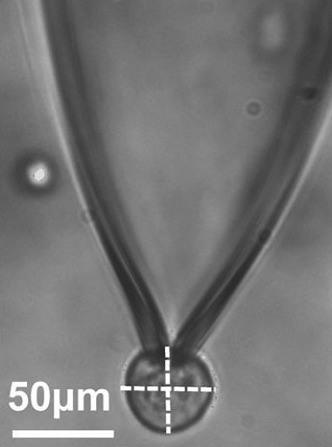

When Robert Hooke first peered down his microscope into a piece of cork, he imagined that the living world was divided into individual cells, like those lived in by the monks of his day (Hooke, 1665). Despite the survival of Hooke’s inapt analogy, the cell is not a static monastery room. The plasma membrane that surrounds the cell can, for example, be absorbed by the cell in a process called endocytosis. A number of different endocytosis pathways have been identified by researchers (Doherty and McMahon, 2009), and with more than 80,000 papers published on the subject, it is surprising that is something left to be categorized. Now, however, in a pair of papers in *eLife*, Donald Hilgemann of the University of Texas Southwestern Medical Center (UTSW) and co-workers describe the mechanisms responsible for a form of endocytosis in which about half of the plasma membrane is absorbed by the cell (Hilgemann et al., 2013; Lin et al., 2013). This form of endocytosis, which is called massive endocytosis, was discovered at UTSW a few years ago (Fine et al., 2011; Hilgemann and Fine, 2011).

Lipid bilayer membranes are nature’s choice for defining temporary borders. The walls of animal cells are downright thin, comprised of lipid bilayers only 40 Å across, and they form all manner of shapes along energetically defined contours. Endocytosis requires energy, and the creation of a closed spherical vesicle from a flat bilayer takes an estimated 200–250 kcal per mole of vesicle (Helfrich, 1973). The most well-established form of endocytosis involves a scaffold protein called clathrin coupling to the membrane, with the energy needed to drive the process coming from ATP (Kirchhausen, 2012), but a number of other mechanisms have been discovered. Endocytosis is studied by the many forms of light microscopy, and in greater detail by electron microscopy, but no single method directly assays all aspects of the process as it occurs in native membranes.

One live assay of endocytosis is called capacitance recording, which is an extension of patch clamp methodology. This technique allows extremely small changes in the area of the plasma membrane (∼0.01 µm^2^) to be detected as individual events and precise measurements to be made of net membrane loss or uptake during exocytosis or endocytosis (Neher and Marty, 1982). Now, in a refreshingly original study, Donald Hilgemann and co-workers at UTSW and Cornell—Michael Fine, Maurine Linder, Benjamin Jennings and Mei-Jung Lin—have used a combination of capacitance recording and other techniques to study the mechanism responsible for the massive endocytosis that occurs after a sudden influx of calcium ions into cells called BHK fibroblasts (Hilgemann et al., 2013).

Massive endocytosis is preceded by depolarization of the inner membrane of the mitochondria and/or the formation of pores called permeability transition pores: both of these processes allow coenzyme A (a small molecule i.e., required for the oxidation of pyruvate and fatty acids) to move from inside the mitochondria to the cytoplasm of the cell. Experimentally, this is accomplished by various mitochondrial insults, such as increasing the concentration of calcium ions inside the cell or poisoning the electron transport chain. Inhibition of calcium uptake blocks massive endocytosis.

The key finding is that the movement of coenzyme A (CoA) into the cytoplasm leads to the synthesis of acyl-CoA, which acts as a substrate for an enzyme, DHHC5, that transfers the fatty acid group called palmitoyl to proteins and thus anchors these proteins to the plasma membrane. In short, mitochondrial damage and the increased availability of substrates for DHHC5 unleash the proteins that initiate endocytosis. One such protein, flotillin, was previously shown to be palmitoylated by DHHC5 (Li et al., 2012). However, proteins are not the only participants in massive endocytosis: cholesterol and PIP_2_ (a phospholipid i.e., found in the cell membrane) can also induce massive endocytosis minutes after the sudden increase in the concentration of calcium ions has subsided (Lariccia et al., 2011).

In the second paper, Hilgemann, Lin, Fine and other co-workers—Jui-Yun Lu and Sandra Hoffman (both UTSW) and Gary Frazier (University of Texas at Dallas)—investigate reperfusion injury, the damage caused when blood supply returns to a tissue after a period of ischemia, in cardiac muscle cells (Lin et al., 2013; Figure 1). Reoxygenation after anoxia, such as occurs during a heart attack, results in massive endocytosis. Using protocols that raise the levels of calcium or acyl-CoA inside the cell, the UTSW team infers that opening the permeability transition pores, activating protein kinase C, and triggering a calcium-dependent mechanism can all induce massive endocytosis. However, massive endocytosis is strongly inhibited in mice lacking DHHC5, resulting in significantly preserved right ventricular contractile function. Finally, Lin et al. show that a diverse range of protein targets undergo palmitoylation (that is, attachment of the fatty acid group, palmitoyl) after reperfusion injury. This most consequential finding of a reduction of massive endocytosis and the preservation of contractility in DHHC5 knockout mice suggests a potential new approach to ameliorate the problem of reperfusion injury.

**Figure 1.**
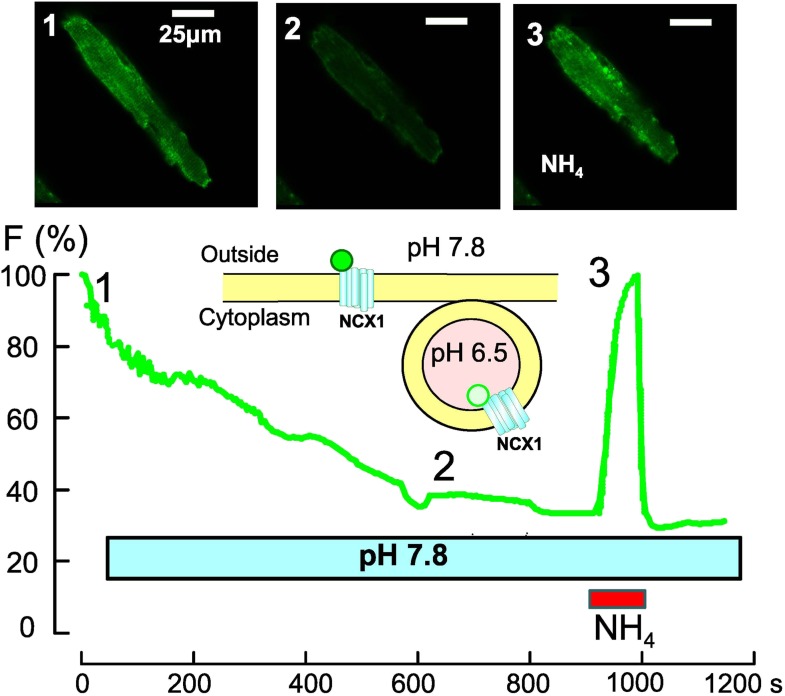
Optical recording of spontaneous massive endocytosis in an isolated cardiac muscle cell. To follow the changes that take place in the cell membrane during massive endocytosis, a Na/Ca exchanger (NCX1) is fused with a pH-sensitive green protein (green circle; Lariccia et al., 2011) and overexpressed in cardiac muscle cells (unpublished data courtesy of Donald Hilgemann). The fluorescence is bright when the green protein is on the external membrane of the cell, where the pH is 7.8 (top left, 1), but it gradually declines during endocytosis as the NCX1 is progressively internalized into vesicles, where the pH is 6.5 (middle, 2). The fluorescence can be increased again by using ammonium chloride (NH_4_Cl) to increase the pH inside the vesicles (right, 3). This shows that massive endocytosis can be activated during routine cell isolation procedures.

D HILGEMANN, UTSW

Although Hilgemann and co-workers tip their hat to the possibility that massive endocytosis can be an ongoing process, the hypothesis mainly rests on dire cellular Ca^2+^ leak conditions, such as those that occur with membrane damage. This sort of large cytoplasmic calcium entry is widely associated with fast massive exocytosis, presumably required for the resealing of membranes (McNeil and Steinhardt, 2003). The link between initial exocytosis followed by endocytosis via vesicles larger than coated vesicles was described in pituitary cells (Thomas et al., 1994). As in those cells, exocytosis in fibroblasts occurs in the first few seconds after a large calcium increase in fibroblasts and is followed by massive endocytosis, but the initial exocytosis is not observed in cardiac muscle cells (Lin et al., 2013). This suggests that these cells lack the pool of vesicles used in the fast exocytosis phase. The identities of these exocytotic or endocytic vesicles are not established.

Other questions remain. Is massive endocytosis an evolutionarily primitive form of endocytosis—the mother of all endocytosis—that separates protein- and cholesterol-rich membranes? Does coenzyme A evoke a regulatory network linking mitochondria to numerous cytoplasmic processes? The understanding of massive endocytosis would be solidified by live tissue imaging of acyl-CoA levels or DHHC5 activity, but these will require the invention of new indicator methods.

One mystery is the role of protein kinase C in massive endocytosis. Since this enzyme phosphorylates phospholemman (a small membrane protein i.e., involved in ion transport), the question arises as to the functional consequence of phosphorylation before or after palmitoylation. Comparison of all palmitoylated proteins before and after massive endocytosis, and the examination of potential newly formed membrane phases, will help illuminate why such massive endocytosis occurs. Perhaps even more important is to follow the fate of such cells over longer durations—that is, is massive endocytosis simply a cellular death throe, or is it a useful adaptation?
